# A case of enterobiasis presenting as post-traumatic-stress-disorder (PTSD): a curious case of the infection with predominant mental health symptoms, presenting for the first time in the settings of a refugee camp

**DOI:** 10.11604/pamj.2017.27.111.12870

**Published:** 2017-06-13

**Authors:** Georgios Karamitros, Nikolaos Kitsos, Fotios Athanasopoulos

**Affiliations:** 1 Medical School, Aristotle University of Thessaloniki, Thessaloniki, Greece; 2 Faculty of Medicine, University of Southampton, Southampton, United Kingdom; 3 Medical School, University of Patra, Patra, Greece

**Keywords:** Enterobiasis, oxyuriasis, enterobius vermicularis, helminth, refugees

## Abstract

Enterobiasis (oxyuriasis) is a common infection in human caused by *Enterobius vermicularis* (*E. vermicularis*), a human intestinal helminth. Because of the easy way of its transmission among people, it has an extremely high prevalence in overcrowded conditions, such as nurseries and primary schools. Oxyuriasis's symptoms are extremely diverse in children, ranging from nausea, diarrhea, insomnia, irritability, recurrent cellulitis, loss of appetite, nightmares and endometritis. Here we report a curious case of oxyuriasis in the settings of a refugee camp in Greece. The patient was a 10-year old Syrian female, who presented with unusual and vague symptoms like insomnia and irritability. Given the violent background of the Syrian warzone that the patient had escaped, she was firstly diagnosed with post traumatic stress disorder (PTSD) before eventually getting correctly diagnosed with enterobiasis. This infection is the first documented case of enterobiasis in the settings of a refugee camp and can highlight the unsanitary living conditions that refugees have to endure in those camps.

## Introduction

Enterobiasis (oxyuriasis) is a common infection in human caused by Enterobius vermicularis (E. vermicularis), a human intestinal helminth [1]. The most effective way of transmission of E. vermicularis is the direct contact between infected and non-infected people [2]. In most cases people acquire enterobiasis via the ingestion of eggs, especially in children contamination of their fingers happens usually because of the direct contact with their anus [3]. Because of the extremely easy way of its transmission among people, it has an extremely high prevalence in overcrowded conditions, such as nurseries and primary schools [4]. The prevalence of E vermicularis is evident around the world and especially in developing countries [5-7]. Oxyuriasis is mostly asymptomatic in adults, however its symptoms are extremely diverse in children, ranging from nausea, diarrhea, insomnia, irritability, recurrent cellulitis, loss of appetite, nightmares and endometritis [8, 9]. In extreme cases E. vermicularis can penetrate the submucosa layer of the bowel and can be fatal [10]. After systematic review of the current literature, we did not find any other published case reports indicating the outbreak of enterobiasis in refugees living in refugee camps. Given the versatility of the epidemiology of the refugee camp and the well established difficulties in health provision in the settings of the refugee camp [11], we were motivated to this case study.

## Patient and observation

We report a case of oxyuriasis that took place in the refugee camp of Alexandria, Greece. The refugee camp hosts about 390 immigrants originated mostly from Syria and secondary from Iran and Pakistan. The sanitary conditions of the camp are below average thus providing the background for the development of infectious diseases and parasites such as the E. vermicularis. A 10-year-old girl from Syria with free medical history, presented to our clinic in mid October, along with her father. The girl did not speak English or Greek, so the communication was very difficult. Finally we managed to find out from her father that the girl suffered from insomnia which started five days ago along with mild diffuse abdominal pain. Her father mentioned that she had lost her appetite. The girl suffered from nausea and vomiting, she was subfebrile (37.2o C) and her abdomen was slightly sensitive. Through her clinical examination we could witness that she was severely malnourished and according to her father she had lost 3 kilos. During the physical examination the abdomen was soft and slightly painful, especially in the lower right quadrant. Mcburney sign was negative. Ultrasonographically, no pathogical signs were found. We also carried out laboratory tests. The complete blood count showed normal Hb, with slight elevation of white blood cell count and eosinophilia (2x109/L). Urine test (stick and culture), as well as, blood glucose level were within normal limits. Because of the nature of the symptoms of the girl, she has been assigned to a children psychologist.

Her symptoms of insomnia, irritability, food avoidance, decreased appetite and the violent background of war that she escaped in Syria seemed to indicate a mental disorder, most likely post traumatic stress disorder (PTSD). However, after a few days the girl and her father came back to the clinic in the refugee camp complaining that her symptoms have gotten a lot worse. This time, beside the sensitivity and the mild diffuse pain in the lower abdomen, the girl complained for itching at the perianal area. The clinical examination of the girl was within normal limits (parameters); however the examination of the anal area showed scars from the scratches. The abovementioned findings suggest that the possibility of oxyuriasis was high. A piece of clear adhesive tape was used to collect a specimen from the perianal surface of the girl, for three consecutive mornings. The samples were mounted (adhesive side down) on a glass slide and screened under a light microscope using 109 and 409 magnifications by expert infectious diseases specialists and microbiologists in the AHEPA Hospital in Thessaloniki, Greece. The results of the microscopic examination showed the presence of oval shaped eggs, which is typical for the specific helminth. Samples were also received from the rest of their family members which were negative. With the diagnosis of Enterobiasis, the girl received the appropriate treatment, which consisted of two doses of albendazole, with each dose two weeks apart, in order to avoid reinfection. The post-treatment period was uneventful. After a 3 month follow-up period, the patient remains asymptomatic without any signs of recurrence.

## Discussion

There is much research on the prevalence of enterobius vermicularis on children. [1, 12, 13] However, data that correlate the prevalence of this helminth on children living in refugee camps were nonexistent until today. Generally, oxyuriasis is an infection that occurs predominantly in areas and communities where the socioeconomic and environmental conditions are way below average, while the hygiene practice levels are low. In our setting, the refugee camp, despite the efforts of volunteers and personnel, the quality of living conditions are in the best case scenario characterized as borderline. These adverse living standards of the refugee camp have been identified by other research papers as well [11]. The aforementioned conditions and practices create the perfect background for the E vermicularis infection. Overcrowded settings and living in non-apartment shelters as well as the unsanitary conditions and failure to wash hands before meals are some significant factors that can increase the risk of acquiring enterobiasis [14]. Although, in our case, the child that eventually diagnosed with oxyuriasis did not have the typical symptoms and the patient initially presented with mental health symptoms like irritability, insomnia, decreased appetite and food avoidance which had never been described in oxyuriasis. Additionally, given the social background of the patient it was only natural for us to make the assumption that the patient suffered from a mental disorder and diagnose her with PTSD. It is well-mentioned in numerous researches that people and especially those who escape war zones and the terrifying images of war, most of the time, suffer from stress, anxiety and depression [11, 15]. In the literature there has been a controversy about which sex ''male or females'' are more susceptible to the parasite infections. Some studies suggest that males are at a higher risk because of their habits and outdoor activities [16] but other studies disagree with those findings [17]. In our case the patient was a female.

**Figure 1:**
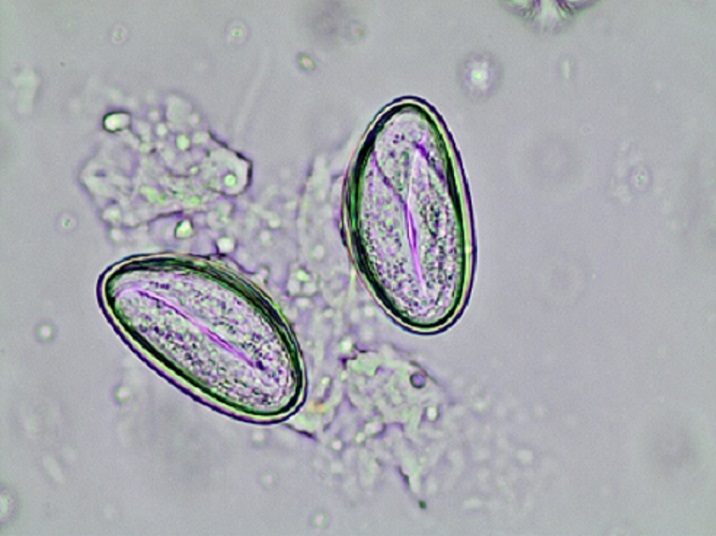
Microscopic caption of enterobious vermicularis eggs

**Figure 2:**
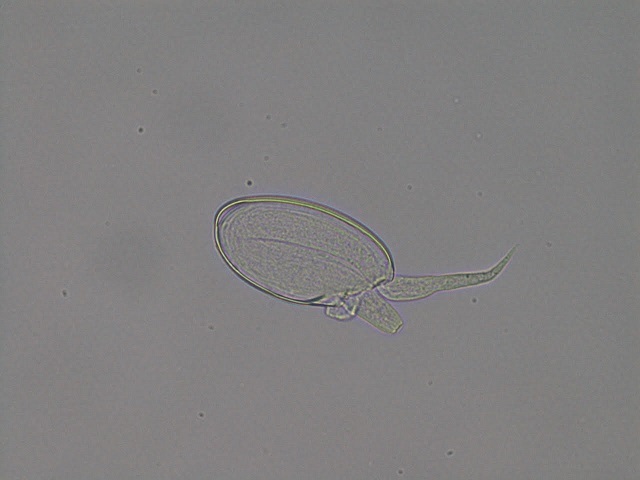
Microscopy of the egg of enterobius vermicularis which is approximately 25x60μm in size

**Figure 3:**
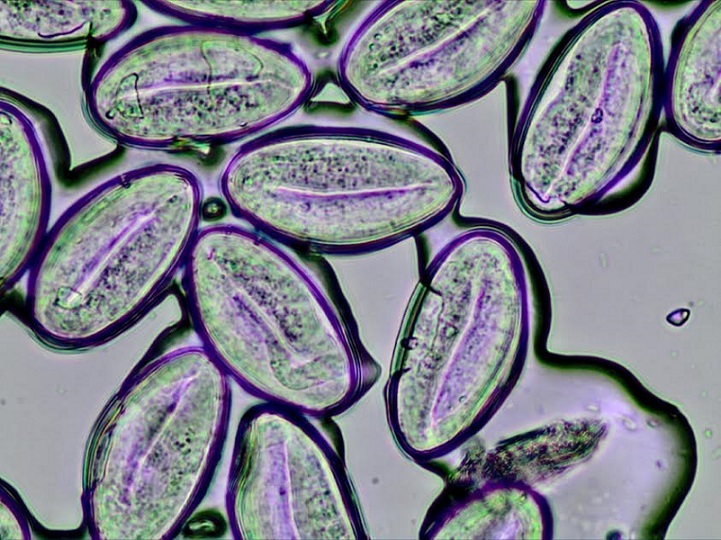
Multiple enterobius vermicularis eggs in a microscopic caption

**Figure 4:**
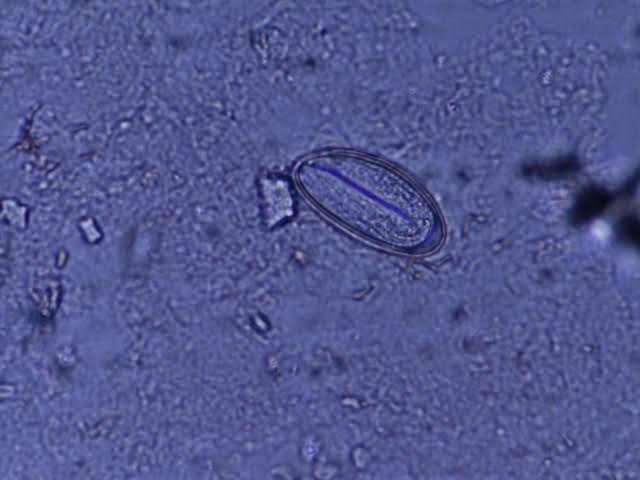
Microscopic image of enterobius vermicularis egg from specimen collected with a clear adhesive tape from the perianal area

**Figure 5:**
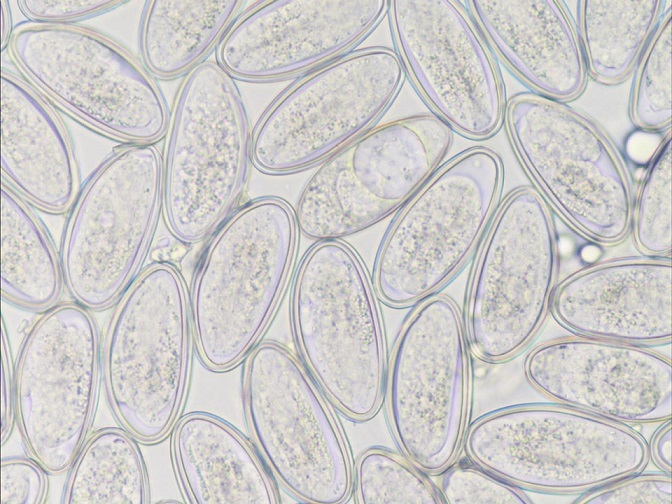
Multiple enterobius vermicularis eggs under microscopic examination

Enterobiasis can be easily transmitted among family members via contaminated hands or inhalation [12], however in our case the family members were healthy and the samples from their adhesive tapes came back negative. Microscopy for direct egg detection in the stool and species identification remains the gold standard and most commonly used technique for the evaluation of intestinal helminth infections [18]. (Figure 1, Figure 2, Figure 3, Figure 4, Figure 5). The other family members remained health and uninfected, most likely because they were educated by the doctors/us to be very cautious and thorough with their personal hygiene in order to avoid the spread of the helminthes. Although, oxyuriasis is not, in general, considered to be a serious disease, there have been described cases of appendicitis that have been attributed to Enterobius vermicularis [19]. And especially in children the morbidity levels of the disease are significant [8]. Thankfully the two doses of albendazole that were prescribed as treatment to the patient were more than enough to counteract the infection. The post-treatment period was uneventful and there were not any complications until the 3-month follow up of the patient. It has been proposed by the World Health Organization (WHO) and there are sufficient studies which suggest that the annual preventive chemotherapy programs against schistosomiasis and soil-transmitted helminthiasis (like oxyuriasis) are cost effective and can radically prevent those diseases in communities with high prevalence and intensity of helminth infections [20]. Many ministries of health in developing countries have implemented the mass drug administration with a single dose of albendazole (400mg) without first establishing a diagnosis for every child. Given the aforementioned data and considering the fact that the majority of the population in a refugee camp comes from developing countries perhaps it would be advisable to implement those programs of mass drug administration in the settings of the refugee camps as well.

## Conclusion

Infection from *E. vermivularis* happens predominantly in children due to poor living conditions and inadequate personal hygiene. This infection is the first documented case of enterobiasis in the settings of a refugee camp and can highlight the unsanitary living conditions that refugees have to endure in those camps. The manifestation of the disease was not typical in our case because it presented mostly with mental symptoms. The initial diagnosis was a false one, because there has been a misinterpretation of the symptoms which were rather vague and general. Doctors and health care providers should always be cautious and consider enterobiasis as an infection in these particular settings.

## Competing interests

The authors declare no competing interest.
